# Comparison of platelet-rich plasma vs hyaluronic acid injections in patients with knee osteoarthritis: A protocol for a systematic review and meta-analysis

**DOI:** 10.1097/MD.0000000000013049

**Published:** 2018-11-02

**Authors:** Yan-hong Han, He-tao Huang, Jian-ke Pan, Jiong-tong Lin, Ling-feng Zeng, Gui-hong Liang, Wei-yi Yang, Jun Liu

**Affiliations:** a Second School of Clinical Medicine, Guangzhou University of Chinese Medicine; b Department of Orthopedics, Second Affiliated Hospital of the Guangzhou University of Chinese Medicine, Guangdong Provincial Hospital of Chinese Medicine; c Bone and Joint Research Team of Degeneration and Injury, Guangdong Provincial Academy of Chinese Medical Sciences, Guangzhou, China.

**Keywords:** hyaluronic acid, knee, meta-analysis, osteoarthritis, platelet-rich plasma, randomized control trials, randomized controlled trials

## Abstract

**Background:**

Knee osteoarthritis (KOA) is a progressive joint disease involving intraarticular and periarticular structures. In recent years, there has been increasing interest in the use of autologous growth factors, such as intraarticular injections of platelet-rich plasma (PRP), to treat KOA. It is necessary to update the research and reevaluate the efficacy and safety of PRP to provide up-to-date evidence for KOA management. Therefore, we provide a protocol for a systematic review of PRP for KOA.

**Methods:**

The aim of this study was to retrieve papers on the topic of PRP treatment for KOA in electronic databases including PubMed, Embase, and the Cochrane Library. The search will include studies that were published from the time the databases were established until April 2018. The entire process will include study selection, data extraction, risk of bias assessment, and meta-analyses.

**Results:**

The literature will provide a high-quality analysis of the current evidence supporting PRP for KOA based on various comprehensive assessments including the Western Ontario and McMaster Universities Osteoarthritis Index, visual analog scale scores, International Knee Documentation Committee scores, Lequesne index scores, and adverse events.

**Conclusion:**

This proposed systematic review will provide up-to-date evidence to assess the effect of PRP treatment for patients with KOA.

**PROSPERO registration number:**

CRD42018108825.

## 1 Introduction

Knee osteoarthritis (KOA) is a progressive joint disease involving intra-articular and periarticular structures.^[1]^ The pathologic characteristics of KOA include articular cartilage lesions, synovitis, subchondral sclerosis, and osteophytosis.^[1]^ Despite advances in medical technology, to date there are no medications or surgical interventions proven to alter the course of KOA development.^[2]^ The current standard of care for patients with symptomatic KOA includes oral anti-inflammatory drugs, physical therapy, topical anti-inflammatory gels, and intra-articular injections.^[2,3]^ Nonsurgical treatments including exercise and weight loss are recommended due to poor symptomatic and functional outcomes with surgical management.^[4]^ However, compliance with nonsurgical treatments is poor, and medications, such as simple analgesics and nonsteroidal anti-inflammatory drugs, are associated with adverse events.^[5,6]^ As reported in many studies and meta-analyses, intra-articular hyaluronic acid (HA) injections are widely used to treat KOA; HA injections are efficacious because the visco-induction properties of HA increase joint lubrication.^[7]^ However, while intra-articular injections of drugs are usually associated with pain relief and increased joint function, they are not effective in patients with severe KOA.^[8]^

During the past decade, there has been increasing interest in the use of autologous growth factors, such as intra-articular injections of platelet-rich plasma (PRP) to treat KOA.^[9]^ Recent research suggests that growth factors and other cytokines released by platelets in response to injury or pathology may modulate inflammatory processes and contribute to the maintenance or regeneration of tissue structures.^[10,11]^ PRP is an autologous blood product that contains an increased concentration of platelets, and it has become an emerging treatment used in orthopedic and sports medicine practices for ligament, tendon, cartilage, and bone injuries.^[12–14]^ As a minimally invasive treatment option, intra-articular injection of PRP has been widely used in clinical therapy. A new technique for the delivery of PRP reported by Sanchez et al^[15]^ is intraosseous infiltration combined with intra-articular injection to treat severe KOA; moreover, no adverse events were reported. A number of randomized controlled trials (RCTs) reported favorable outcomes of PRP injections^[16–18]^; several systematic reviews and meta-analyses have been published that concluded that PRP was an effective and safe orthobiologic to use in the treatment of KOA when compared with other intra-articular injections.^[19–21]^ However, these reviewers did not reach consensus in terms of the effects of PRP on pain relief and functional recovery; they also concluded that more RCTs, in particular high-quality studies, were still needed. Prior reviews either included non-RCTs or only synthesized a small number of RCTs (<10) for analysis, and additional randomized trials have since been published. Therefore, it is necessary to perform an updated systematic review and meta-analysis to fully investigate the temporal effect of PRP on knee pain and physical function. We aimed to identify all prospective, randomized trials published to date to provide the latest insights into the efficacy of the use of PRP for treating KOA.

## 2 Methods

### 2.1 Data sources and search strategy

The study was approved by the ethics committee of Guangdong Provincial Hospital of Chinese Medicine. We will adhere to the Preferred Reporting Items for Systematic Reviews and Meta-analysis (PRISMA) statements for reporting systematic reviews. A comprehensive of the PubMed (1966–April 2018), Embase (1980–April 2018), and Cochrane Library (1966–April 2018) databases will be performed. The search terms will include “platelet-rich plasma,” “PRP,” “knee,” “osteoarthritis,” “arthritis,” and “arthritic.” No language exclusions will be applied. The references of the identified studies will be manually searched.

### 2.2 Inclusion criteria and study selection

#### 2.2.1 Participants

Only published articles enrolling adult participants with a diagnosis of KOA will be included. All participants will be diagnosed with KOA. The patient's gender, age, and grades of KOA will not be limited.

#### 2.2.2 Interventions

The intervention group will have received intraarticular PRP, and there will be no restriction on the dosages of PRP or the time interval between injections.

#### 2.2.3 Comparisons

The control group will have received HA alone.

#### 2.2.4 Outcomes

The primary outcome will be the Western Ontario and McMaster Universities (WOMAC) Osteoarthritis Index. The secondary outcomes will be visual analog scale (VAS) scores, International Knee Documentation Committee (IDKC) scores, Lequesne index scores, and adverse events.

#### 2.2.5 Study design

**Figure 1:**
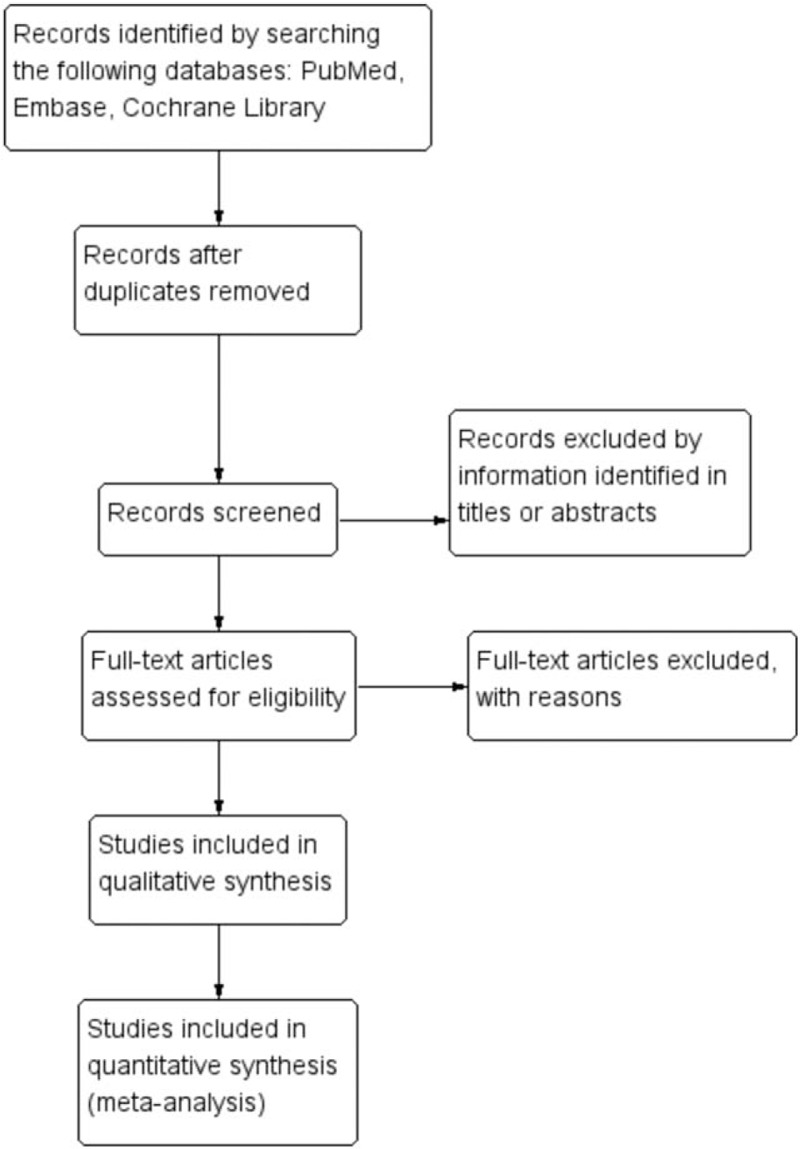
Flow diagram of study selection.

Clinical RCTs will be considered eligible for our study. Articles will be excluded from the current meta-analysis if they are duplicate articles, cohort studies, retrospective studies, case reports, letters, editorials, conference abstracts, or animal experimental studies. The flow diagram of the study selection is shown in Figure 1.

### 2.3 Data extraction

Two reviewers will independently extract the key data from the included studies: first authors’ names, date of publication, sample size, patients’ age and gender, grades of KOA, injection dose, times, intervals of PRP and HA, and follow-up periods. The primary outcome will be the WOMAC osteoarthritis index. The secondary outcomes will be VAS scores, IDKC scores, Lequesne scores, and adverse events. In the event of missing data, we will attempt to contact the corresponding authors for details.

### 2.4 Assessment of methodologic quality

The methodologic quality of the included studies will be independently evaluated by 2 reviewers using the Cochrane Collaboration tool for assessing the risk of bias (ROB). Each study will be reviewed and scored as having a high, low, or unclear ROB according to the following domains: selection bias (random sequence generation and allocation concealment), performance bias (blinding of participants and personnel), detection bias (blinding of outcome assessments), attrition bias (incomplete outcome data), reporting bias (selective reporting), and other bias (other sources of bias). Any discrepancies between the findings of the reviewers will be arbitrated by the corresponding author.

### 2.5 Data analysis and statistical methods

All statistical analyses will be performed using Manager 5.3.5 (Cochrane Collaboration, Oxford, UK) software. Continuous outcomes will be calculated and expressed as the mean difference (MD), and dichotomous outcomes will be expressed as risk ratios (RRs). Heterogeneity between studies will be quantified using the *I*^2^ statistic. We will use an *I*^2^ value of <25% to represent low heterogeneity and an *I*^2^ value of <75% to indicate high heterogeneity. When high heterogeneity exists, random-effects models will be used. The results will be presented as MDs with 95% confidence intervals. A *P*-value of <.05 will be considered statistically significant.

## 3 Discussion

As a chronic progressive joint disease, KOA is the second leading cause of loss of function,^[4]^ and it imposes a heavy economic and social burden.^[22]^ The etiology and pathogenesis of KOA are still not clear^[23]^; the main pathologic change is articular cartilage degeneration with changes in synovial fluid components.^[24]^

As a vector for large growth factors,^[25]^ PRP can promote tissue repair^[26]^ and is increasingly being used in the treatment of KOA. Some reports suggest that PRP may induce the migration, proliferation, and differentiation of precursor cells in the synovial fat pad or cartilage. Therefore, PRP can promote repair of damaged cartilage while reducing pain and the effects of the inflammatory response.^[27]^

Most of the previous systematic reviews and meta-analysis have shown that PRP is an effective and safe alternative treatment for long-term pain relief and functional improvement in patients with KOA. However, the previous conclusions were reached on the basis of the small number of RCTs^[28]^; thus, the temporal effects of PRP injections on knee pain and physical function have not been fully investigated. Chang et al^[29]^ found that patients receiving PRP exhibited better and more prolonged improvement than those receiving HA; however, half of the 16 studies they included in their analysis were case series, and 5 were RCTs. Laudy et al^[30]^ pooled 10 trials, including 6 RCTs and found that PRP injections reduced pain and improved function more effectively than placebo or HA injections in patients with KOA. Nonetheless, most comparisons included only 1 or 2 studies due to the small number of RCTs that were pooled for analysis.

It cannot be denied that a high-quality trial is still lacking; however, clinical studies on this topic have also been published recently. We have reported a protocol for performing a systematic review to provide up-to-date data regarding the effectiveness and safety of PRP for KOA. Therefore, we will begin to conduct the review when the necessary trials are met, and all operating procedures will be performed in accordance of Cochrane Handbook to ensure that the provided information is helpful for clinicians and KOA patients. This review is registered in PROSPERO Registry: CRD42018108825 (https://www.crd.york.ac.uk/PROSPERO/).

## Acknowledgment

The authors thank American Journal Experts for their linguistic assistance during the preparation of this manuscript.

## Author contributions

Conceived and designed the experiments: JL and YHH. Performed the experiments: YHH, JKP, and HTH. Analyzed the data: JTL, LFZ, and GHL. Contributed reagents/materials/analysis tools: YHH, LFZ, HTH, and WYY. Wrote the paper: YHH, JKP, and HTH.

**Conceptualization:** Yan-hong Han, Jun Liu.

**Data curation:** Yan-hong Han, He-tao Huang, Jian-ke Pan.

**Formal analysis:** Yan-hong Han, Jian-ke Pan, Wei-yi Yang.

**Funding acquisition:** Jiong-tong Lin, Ling-feng Zeng, Jun Liu.

**Investigation:** He-tao Huang, Jiong-tong Lin, Gui-hong Liang, Jun Liu.

**Methodology:** Jian-ke Pan, Ling-feng Zeng, Gui-hong Liang, Jun Liu.

**Project administration:** He-tao Huang, Ling-feng Zeng, Gui-hong Liang, Jun Liu.

**Resources:** Jian-ke Pan, Wei-yi Yang.

**Software:** Yan-hong Han, He-tao Huang, Jian-ke Pan, Jiong-tong Lin.

**Supervision:** Gui-hong Liang, Wei-yi Yang.

**Validation:** Wei-yi Yang, Jun Liu.

**Visualization:** Jiong-tong Lin.

**Writing – original draft:** Yan-hong Han, Jian-ke Pan.

**Writing – review & editing:** Jun Liu.
